# Dr. Coleman Nathaniel Clark

**Published:** 1913-03

**Authors:** 


Dr. Coleman Nathaniel Clark, Buffalo, 1865, died of influenza at his home in St. Charles, Minn., January 30, aged 73.

